# Smoking cessation care can translate to lower hazard of death in the short-run in cancer patients - a retrospective cohort study to demonstrate the value of smoking cessation services within the treatment phase of cancer

**DOI:** 10.1186/s12885-019-5778-y

**Published:** 2019-07-01

**Authors:** F. I. Hawari, N. A. Obeidat, D. Rimawi, K. Jamal

**Affiliations:** 1 Section of Pulmonary and Critical Care, Department of Medicine, King Hussein Cancer Center, P.O. Box 1269, Queen Rania Al Abdullah Street, Amman, Al-Jubeiha 11941 Jordan; 2 Cancer Control Office, King Hussein Cancer Center|, Amman, Jordan; 3 Office of Scientific Affairs – Center of Research Shared Resources, King Hussein Cancer Center, Amman, Jordan; 4 Office of Scientific Affairs – Cancer Registry, King Hussein Cancer Center, Amman, Jordan

**Keywords:** Smoking cessation intervention, Treatment, Cancer care, Survival, Mortality

## Abstract

**Background:**

Smoking cessation is a key step towards improving cancer care and outcomes. However, smoking cessation interventions are underprovided in oncology settings. Within Jordan’s only comprehensive oncology center, we sought to evaluate receipt of care at a smoking cessation clinic and the effect of assisted abstinence through the smoking cessation clinic on short-term (two-year) survival after a cancer diagnosis.

**Methods:**

We employed a retrospective cohort study design. Cancer registry and smoking cessation clinic data for adult Jordanian cancer patients diagnosed between 2009 and 2016, who also were cigarette smokers, and who received full treatment at King Hussein Cancer Center, were analyzed. Specifically, descriptive statistics of patients who visited the smoking cessation clinic were generated, and short-term (two-year) hazard of death of patients based on whether or not smoking cessation clinic-assisted abstinence occurred, were evaluated.

**Results:**

There were 3403 patients who met our inclusion criteria. Approximately 21% of cancer patients were seen at the smoking cessation clinic, and significant demographic and clinical disparities in who was being seen [at the smoking cessation clinic] existed. In 2387 patients with available survival data, smokers who never went to the smoking cessation clinic (or were seen only once, or seen a year or more from diagnosis) had a hazard of death 2.8 times higher than smokers who had visited the smoking cessation clinic and who also confirmed they had not smoked on atleast two of their 3-, 6- or 12-month follow-up visits (95% confidence interval [CI] = 1.7–4.6). Non-abstainers at the smoking cessation clinic exhibited a similar disadvantage (HR 2.7, 95% CI 1.4–5.0).

**Conclusions:**

Although evidence-based smoking cessation interventions increase the likelihood of abstinence and can lower the short-term hazard of death during cancer treatment, there is a deprioritization of smoking cessation interventions during cancer care, as indicated by low proportions of patients seen at the smoking cessation clinic. Our findings emphasize the importance of promoting interventions to avail smoking cessation interventions in oncology settings within the cancer treatment phase.

## Background

Given the rising global burden of cancer, ensuring that cancer care is optimized and availed equitably has become an increasingly challenging priority for most countries, and resource constraints have rendered many therapeutic interventions difficult if not impossible to access [1]. In response to this global challenge, evaluating and promoting healthcare interventions that are relatively less resource-intensive and can benefit a broad range of cancer patients has become a pressing matter.

Smoking cessation management for cancer patients offers one such example of a cost-effective intervention to improve patient outcomes, and has been called for by many [2–10]. Conversely, continuing to smoke after a cancer diagnosis negatively impacts cancer treatment and quality of life, and has been associated with shortened survival in the long-term in specific cancers [11–20]. However, despite the clear clinical value of smoking cessation in cancer patients, various studies indicate that smoking cessation interventions are not sufficiently practiced in the oncology setting [21–24]. Thus, more efforts are needed to draw attention to this practice gap, and more importantly to highlight the lost opportunities in improving cancer patient outcomes as a result of this gap.

We were interested in demonstrating the benefits of smoking cessation during cancer treatment by examining the two-year survival benefits associated with smoking cessation clinic-assisted abstinence, among a diverse group of cancer patients. We also were interested in identifying which patient subgroups were less likely to receive smoking cessation care, thereby potentially foregoing the benefits of smoking cessation during cancer treatment. We hypothesized that patients receiving care at the smoking cessation clinic and reporting abstinence would be at survival advantage to cancer patients who did not receive such care. We also hypothesized that being older, having an advanced stage of cancer, and not having a tobacco-related cancer in the conventional sites known to be associated with tobacco use (oropharynx, larynx, esophagus, trachea, bronchus, lungs) were associated with a decreased likelihood of receiving care at the smoking cessation clinic.

## Methods

### Setting

King Hussein Cancer Center (KHCC) in Amman, Jordan is a Joint commission, disease-specific accredited comprehensive cancer center which offers cancer care to a substantial proportion of the Jordanian population, and also serves as a regional cancer treatment hub for the Middle East [25]. Approximately 4350 patients are seen annually, roughly 60% of whom complete treatment at the Center.

### Study design

A retrospective cohort study design was used to analyze hospital-based cancer registry records and clinical records of cancer patients receiving care at KHCC’s smoking cessation clinic.

### Participants

The analysis was based on records from KHCC, and included adult Jordanian cancer patients who were current smokers and receiving care at the Center. Records spanning patients diagnosed between the years 2009 and 2016 were included. Inclusion criteria were being 18 years or older, being a Jordanian, having a documented status as a current [cigarette] smoker in KHCC’s registry records, and receiving complete care at KHCC.

### Variables

#### Outcome variable

Two-year hazard of death after diagnosis was measured to evaluate whether or not visiting the smoking cessation clinic within a year of diagnosis (when most interventions to treat patients are undergone) imparted survival benefits (presumably by inducing greater rates of abstinence among those cancer patients visiting the clinic than cancer patients not receiving specialized cessation care). Reliable survival data were available from years 2012 onwards at the Center. Survival analyses were therefore conducted on a subgroup of patients (*n* = 2387, those diagnosed after 2011).

An additional variable, whether or not a patient was seen at the smoking cessation clinic, was analyzed as an outcome variable in order to identify which patient subgroups were at higher risk of not receiving smoking cessation care.

#### Independent variable

With regards to predictors associated with two-year survival, we were interested in ascertaining whether or not a survival advantage may have been observed in patients visiting the smoking cessation clinic (as a result of their abstinence due to the receipt of smoking cessation care). The following section describes the possible profiles for our sample of cancer patients [who smoked]:

- Patients who visited the smoking cessation clinic: Generally, patients who visit the cessation clinic are followed to record 3-, 6- and 12-month repeated seven-day point-prevalence [self-reported] abstinence status, a measure that is used in the smoking cessation literature [26, 27]. Specifically, at each follow-up point, patients are asked if they smoked in the past 7 days. Thus, within this category of patients who visited the clinic, patients were further categorized as follows:
  - Patients with two or more follow-up points (from a maximum of three follow-up points) indicating positive point-prevalence abstinence (1)
  - Patients with only one follow-up point (from a maximum of three follow-up points) indicating positive point-prevalence abstinence (2)
  - Patients with more than one follow-up point (from a maximum of three follow-up points) all indicating negative point-prevalence abstinence (3)
  - Patients visiting the smoking cessation clinic more than once [within a year of diagnosis] but not having accrued sufficient follow-up information (4)
  - Patients who visited the clinic only once and did not return to the clinic (5)
  - Patients who came to the clinic after a year of diagnosis (6)
- Patients who never visited the smoking cessation clinic (7)

According to the above profiles, four main groupings of patients were created to describe abstinence trajectories:

- **Group A** (equivalent to 1 above): Patients who were seen [within a year of diagnosis] more than once at the clinic and whose records indicated abstinence at atleast two follow-up points from a maximum of three follow-up points (3-, 6- and 12-months)
- **Group B** (equivalent to 2 above): Patients who were seen [within a year of diagnosis] more than once at the clinic and whose records indicated abstinence at only one follow-up point from a maximum of three follow-up points;
- **Group C** (equivalent to 3 or 4 above): Patients who were seen [within a year of diagnosis] more than once at the clinic and whose records indicated no abstinence at any follow-up point. Patients who did not accrue sufficient follow-up records to measure abstinence also were included in this group (i.e. assumed to be non-abstinent);
- **Group D** (equivalent to 5, 6, or 7 above): Patients who were never seen at the clinic or seen only once, or were seen after a year from diagnosis.

#### Other covariates

various sociodemographic and clinic variables were included as control variables given their potential for being associated with our outcome variables of interest. These specifically included patient demographics (age at diagnosis, gender, marital status, and geographic area of original residence – defined as North, Central and South in Jordan); diagnostic clinical variables (cancer primary site; whether or not a cancer was tobacco-related; stage of cancer; and year of diagnosis); treatment prescribed (chemotherapy, radiation, surgery, immunotherapy, hormonal therapy, bone marrow transplant); and comorbidities (diabetes, respiratory disease, and cardio- or cerebrovascular disease).

### Analysis

Descriptive and multivariable analyses were first run to compare the characteristics of patients visiting the smoking cessation clinic with those who never visited the clinic. Specifically, multivariable logistic regression was performed to determine which demographic of clinical factors were significant predictors of whether or not a cancer patient received smoking cessation care.

In order to ascertain survival benefits possibly associated with becoming abstinent through the smoking cessation clinic, survival curves were compared across the four groups of patients described earlier (Groups A through D). A multivariable Cox regression was performed to determine whether or not the various patient profiles were significantly associated with survival benefits, controlling for demographic, geographic and clinical factors.

## Results

From 14,813 registry records, we were able to identify 3403 unique Jordanian cancer patients documented as current smokers. A descriptive table of the patient population, categorized by whether or not they received smoking cessation care at the clinic, is provided in Table 1. Approximately 21% of patients (717) were seen atleast once at the smoking cessation clinic. At the bivariate level, females, the oldest age group, and those who did not have tobacco-related solid tumors in the head and neck area or lungs, were less likely to be seen at the clinic (than males, the youngest age group, and those who had tobacco-related solid tumors in the head and neck area or lungs, respectively). Those diagnosed in 2015 to 2016 (relative to 2009) and those with an in-situ staging (relative to local stage) were more likely to be seen at the clinic (Table 1).

**Table 1:** Demographic and clinical characteristics associated with the odds of being seen at the smoking cessation clinic at KHCC in a sample of 3403 Jordanian cancer patients who smoke (column totals presented). A total of 717 cancer patients were seen at the clinic while 2686 were not

Variable:	Proportion seen at the clinic (*n*)	Unadjusted odds ratio of being seen (95% CI)	Adjusted odds ratio (of being seen)
Gender			
Males (*N* = 2454)	23.5% (*n* = 576)	Reference group	Reference group
Females (*N* = 949)	14.9% (*n* = 141)	0.59 (0.46–0.70)	0.49 (0.38–0.63)
Age at diagnosis			
18–30 (*N* = 278)	21.9% (*n* = 61)	Reference group	Reference group
31–40 (*N* = 491)	17.5% (*n* = 86)	0.76 (0.52–1.1)	0.89 (0.57–1.4)
41–50 (*N* = 776)	24.4% (*n* = 189)	1.1 (0.83–1.6)	1.3 (0.82–1.9)
51–60 (*N* = 860)	24.2% (*n* = 208)	1.1 (0.82–1.6)	1.1 (0.73–1.7)
61–70 (*N* = 675)	19.1% (*n* = 129)	0.84 (0.60–1.2)	0.78 (0.49–1.2)
> 70 (*N* = 323)	13.6% (*n* = 44)	0.56 (0.37–0.86)	0.49 (0.29–0.85)
Diagnosis year			
2009 (*N* = 256)	14.8% (*n* = 38)	Reference group	Reference group
2010 (*N* = 382)	13.1% (*n* = 50)	0.86 (0.55–1.4)	0.85 (0.53–1.4)
2011 (*N* = 378)	14.3% (*n* = 54)	0.96 (0.61–1.5)	0.94 (0.60–1.5)
2012 (*N* = 329)	13.4% (n = 44)	0.89 (0.55–1.4)	0.86 (0.53–1.4)
2013 (*N* = 416)	15.9% (*n* = 66)	1.1 (0.70–1.7)	1.0 (0.66–1.6)
2014 (*N* = 492)	20.7% (*n* = 102)	1.5 (0.99–2.3)	1.4 (0.91–2.1)
2015 (*N* = 572)	28.5% (*n* = 163)	2.3 (1.5–3.4)	2.4 (1.6–3.6)
2016 (*N* = 578)	34.6% (*n* = 200)	3.0 (2.1–4.5)	3.3 (2.2–4.9)
Marital status			
Married (*N* = 2825)	21.3% (*n* = 601)	Reference group	Reference group
Single (*N* = 378)	18.5% (*n* = 70)	0.84 (0.64–1.1)	0.88 (0.62–1.3)
Separated/divorced/widowed (*N* = 200)	23.0% (*n* = 46)	1.1 (0.79–1.6)	1.4 (0.93–2.0)
Geographic area			
Central (*N* = 2860)	21.4% (*n* = 613)	Reference group	Reference group
North (*N* = 350)	19.1% (*n* = 67)	0.87 (0.66–1.1)	0.74 (0.55–0.99)
South (*N* = 192)	19.3% (*n* = 37)	0.88 (0.60–1.3)	0.80 (0.55–1.2)
Tumor type and relation to tobacco			
Solid, tobacco-related^a^ (*N* = 851)	25.5% (*n* = 217)	Reference group	Reference group
Solid, tobacco-related (other)^b^ (*N* = 827)	20.1% (*n* = 166)	0.73 (0.58–0.92)	0.57 (0.43–0.75)
Solid, not tobacco-related (*N* = 1186)	16.8% (*n* = 199)	0.59 (0.48–0.73)	0.51 (0.38–0.69)
Hematological, tobacco-related (*N* = 60)	13.3% (*n* = 8)	0.45 (0.21–0.96)	0.52 (0.23–1.2)
Hematological, not tobacco-related (*N* = 478)	26.6% (*n* = 127)	1.1 (0.82–1.4)	1.1 (0.8–1.5)
Stage			
In-situ (*N* = 107)	29.9% (*n* = 32)	1.6 (1.03–2.5)	1.7 (1.03–2.8)
Local (*N* = 797)	20.8% (*n* = 166)	Reference group	Reference group
Regional (*N* = 1229)	22.8% (*n* = 280)	1.1 (0.90–1.4)	0.95 (0.74–1.2)
Distant or unstaged (*N* = 1270)	18.8% (*n* = 239)	0.89 (0.71–1.1)	0.54 (0.40–0.71)
Received chemotherapy			
No (*N* = 1027)	19.6% (*n* = 201)	Reference group	Reference group
Yes (*N* = 2376)	21.7% (*n* = 516)	1.14 (0.95–1.5)	1.4 (1.1–1.8)
Received surgery			
No (*N* = 2153)	21.0% (*n* = 451)	Reference group	Reference group
Yes (*N* = 1250)	21.3% (*n* = 266)	1.0 (0.86–1.2)	1.0 (0.80–1.3)
Received radiation			
No (*N* = 1867)	21.2% (*n* = 395)	Reference group	Reference group
Yes (*N* = 1536)	21.0% (*n* = 322)	0.99 (0.84–1.2)	0.96 (0.78–1.2)
Received hormonal therapy			
No (*N* = 2944)	21.8% (*n* = 643)	Reference group	Reference group
Yes (*N* = 459)	16.1% (*n* = 74)	0.69 (0.53–0.90)	1.3 (0.94–1.9)
Received immunotherapy			
No (*N* = 3331)	20.9% (*n* = 696)	Reference group	Reference group
Yes (*N* = 72)	29.2% (*n* = 21)	1.6 (0.93–2.6)	1.6 (0.92–3.0)
Received BMT			
No (*N* = 3283)	20.9% (*n* = 687)	Reference group	Reference group
Yes (*N* = 120)	25.0% (*n* = 30)	1.3 (0.83–1.9)	1.6 (0.99–2.5)
Have diabetes			
No (*N* = 2826)	20.7% (*n* = 586)	Reference group	Reference group
Yes (*N* = 577)	22.7% (*n* = 131)	1.1 (0.91–1.4)	0.99 (0.77–1.3)
Have respiratory disease			
No (*N* = 3122)	20.9% (*n* = 653)	Reference group	Reference group
Yes (*N* = 281)	22.8% (*n* = 64)	1.1 (0.83–1.5)	0.98 (0.70–1.4)
Have cardio- or cerebrovascular disease			
No (*N* = 2464)	20.5% (*n* = 506)	Reference group	Reference group
Yes (*N* = 939)	22.5% (*n* = 211)	1.1 (0.94–1.3)	1.1 (0.90–1.4)

^a^ Head, neck, respiratory, esophageal

^b^ Colorectal, cervical, pancreatic, urinary, stomach and liver

*p* < 0.05

When a multivariable logistic regression was conducted to determine significant predictors of being seen at the clinic (Table 1), females, patients in the oldest age group (relative to the youngest one), patients residing in the North (relative to living in Central Jordan), patients having solid tumors that were not tobacco-related (i.e. tobacco-related solid tumors in sites distant to the respiratory or head and neck area), and patients having an advanced stage of cancer (relative to having a localized stage) were significantly less likely to be seen at the clinic. Those diagnosed in 2015–2016 (relative to 2009), those with an in-situ staging (relative to local stage), and those receiving chemotherapy, were significantly more likely to be seen at the clinic.

### Value of receiving smoking cessation care

**Fig. 1:**
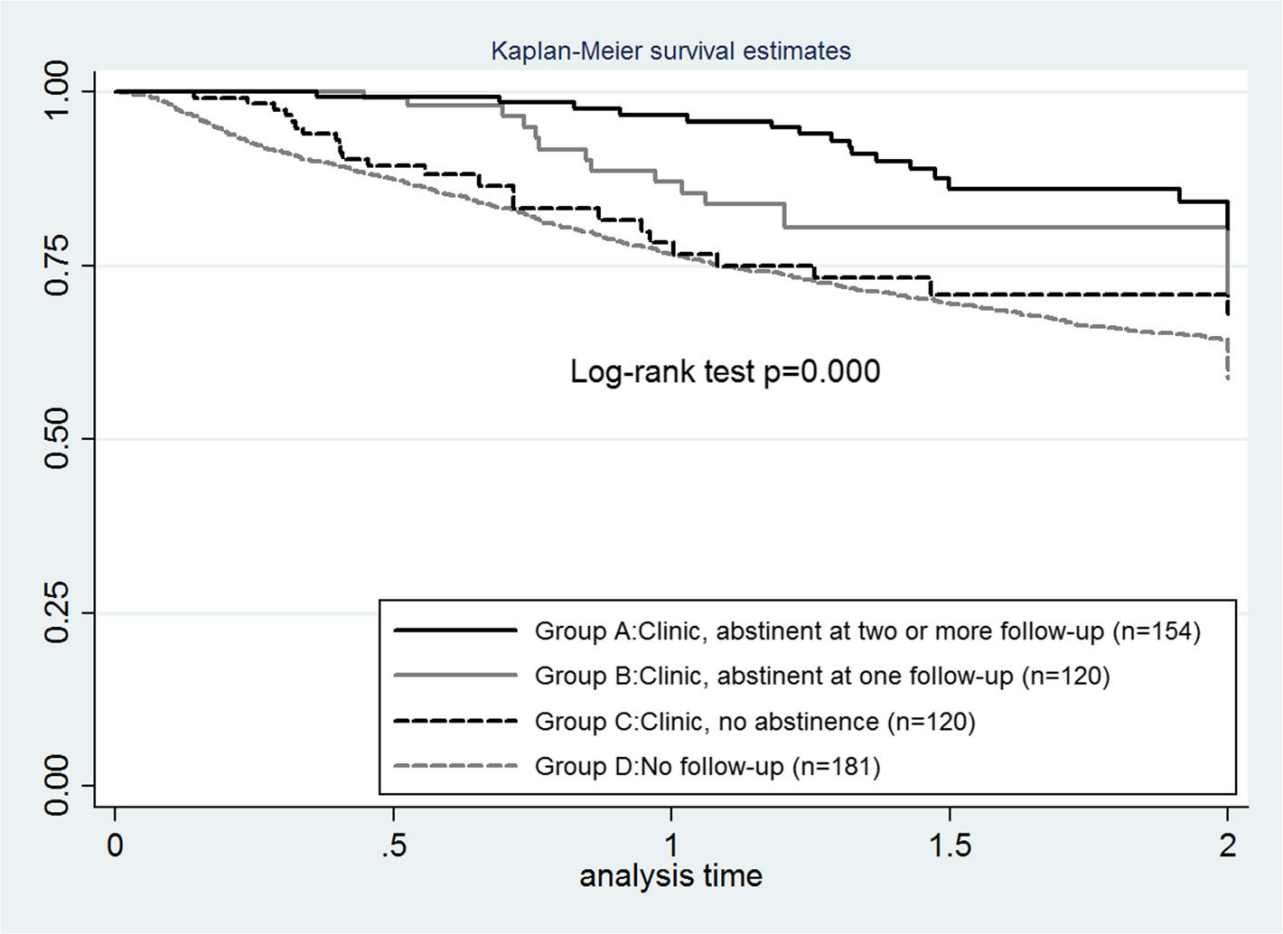
Survival curves for a subsample of cancer patients with survival data (*N* = 2387) divided as follows: smokers who were seen [within a year of diagnosis] more than once at the clinic and whose records indicated abstinence at atleast two follow-up points (group A); smokers who were seen [within a year of diagnosis] more than once at the clinic and whose records indicated abstinence at only one follow-up point (group B); smokers who were seen [within a year of diagnosis] more than once at the clinic and whose records indicated no abstinence at any follow-up point. Patients who did not accrue sufficient follow-up records to measure abstinence also were included in this group (group C); and smokers who were never seen at the clinic or were seen after a year from diagnosis (group D)

Within the subsample of 2387 patients included in the survival analysis, Kaplan-Meier survival curves across the different patient groups are displayed in Fig. 1, and demonstrate a survival advantage for smokers in Group A (patients seen more than once at the smoking cessation clinic and whose records indicated abstinence on atleast two of their 3-, 6- or 12-month follow-up visits). In addition, a dose effect was observed: smokers in Group B (those seen more than once at the clinic and whose records indicated abstinence at only one follow-up point from a maximum of three follow-up points) had a better survival prognosis than smokers in Group C (those seen more than once at the clinic and whose records indicated no abstinence at any follow-up) and smokers in Group D (those never seen at the clinic or seen only once, or were seen after a year from diagnosis). The survival advantages observed on a bivariate level persisted after running a multivariable Cox regression analysis using Group A (patients seen more than once at the smoking cessation clinic and whose records indicated abstinence on atleast two of their 3-, 6- or 12-month follow-up visits) as a reference group, and controlling for other demographic and clinical factors that influence patient survival (Table 2). After adjustments, patients in Group D (those never seen at the clinic or seen only once, or were seen after a year from diagnosis) had a hazard of death 2.8 times higher than the reference group (95% confidence interval, CI, 1.7–4.6). Non-abstainers at the smoking cessation clinic (Group C) also exhibited a similar disadvantage (HR 2.7, 95% CI 1.4–5.0) relative to Group A.

**Table 2:** Multivariable Cox proportional hazards regression analysis of factors associated with two-year mortality of Jordanian cancer patients at KHCC who smoke (*n* = 2384)

Variable	HR (95% CI)
Patient group (with regards to being seen at cessation clinic)	
Smokers who were seen [within a year of diagnosis] more than once at the clinic and whose records indicated abstinence at atleast two follow-up points (group A)	Reference group
Smokers who were seen [within a year of diagnosis] more than once at the clinic and whose records indicated abstinence at only one follow-up point (group B)	1.3 (0.65–2.6)
Smokers who were seen [within a year of diagnosis] more than once at the clinic and whose records indicated no abstinence at any follow-up point (group C)	2.7 (1.4–5.0)*
Smokers who were never seen at the clinic or were seen after a year from diagnosis (group D)	2.8 (1.7–4.6)*
Female (versus male)	0.67 (0.53–0.84)*
Marital status	
Married	Reference group
Single	0.88 (0.62–1.2)
Other	1.1 (0.74–1.6)
Age at diagnosis	
18–30	Reference group
31–40	1.5 (0.91–2.4)
41–50	1.6 (0.98–2.6)
51–60	1.8 (1.1–2.9)*
61–70	2.1 (1.3–3.5)*
> 70	2.1 (1.2–3.5)*
Year of diagnosis	
2012	Reference group
2013	0.83 (0.66–1.0)
2014	0.84 (0.66–1.1)
2015	0.89 (0.70–1.1)
2016	0.72 (0.52–0.99)*
Geographic area	
Central	Reference group
North	1.1 (0.83–1.4)
South	0.91 (0.65–1.3)
Cancer site (type)	
Solid, tobacco-related: head, neck, respiratory, esophageal (reference group)	Reference group
Solid, tobacco-related, other: cervical, pancreatic, liver, stomach, urinary	0.75 (0.60–0.93)*
Solid, not tobacco-related	0.92 (0.72–1.2)
Hematological, tobacco-related	0.76 (0.47–1.2)
Hematological, not tobacco-related	0.20 (0.14–0.27)*
Stage	
Local (reference group)	Reference group
In-situ	0.37 (0.10–1.6)
Regional*	3.7 (2.5–5.4)*
Distant and unstaged	13.1 (9.0–19.1)*
Received chemotherapy (yes/no)	0.49 (0.39–0.61)*
Received surgery (yes/no)	0.39 (0.31–0.50)*
Received radiation (yes/no)	0.73 (0.60–0.87)*
Received immunotherapy (yes/no)	0.81 (0.29–1.3)
Received hormonal therapy (yes/no)	0.18 (0.11–0.28)*
Received bone marrow transplant (yes/no)	0.46 (0.27–0.77)
Having diabetes	1.2 (0.94–1.4)
Having cardio- or cerebrovascular disease	0.99 (0.81–1.2)
Having respiratory disease	1.2 (0.89–1.5)

*p < 0.05

## Discussion

Our study sought to demonstrate the value of clinic-assisted abstinence in a diverse cancer patient population in Jordan, and to provide insight into which patient subgroups were at higher risk of not being seen at the smoking cessation clinic.

When survival of cancer patients who received smoking cessation care was compared to that of patients who did not receive any care, our results indicated that patients who did not receive care at the smoking cessation clinic and those who did not report any abstinence despite visiting the clinic fared significantly more poorly (in terms of two-year mortality) than those who visited the clinic and reported being abstinent at more than follow-up point. The advantage in the lattermost group persisted after running a multivariable Cox proportional hazards regression analysis adjusting for sociodemographic and clinical factors that may also have impacted hazard of death. While our data did not measure the specific pathways by which survival benefits were accrued during receipt of smoking cessation services, it is plausible that the short-run survival benefit may be explained by the averted many risks of continued smoking among cancer patients (poorer response to chemotherapy; inferior bone marrow transplantation outcomes; higher needs for critical care admissions; and greater risks of treatment toxicities, surgical complications, infections, and recurrence) [14, 28–33]. In addition, we observed a survival advantage for those who reported abstinence at only one follow-up point (Group B), which may indicate a harm reduction effect.

While most studies have focused on the positive impact of smoking cessation on long-term survival in cancer patients, our findings reveal significant differences in hazard of death within the two-year period after diagnosis, in a cancer patient sample that included various sites and stages; and imply that integrating specialized smoking cessation interventions to induce smoking abstinence during the crucial period of cancer treatment can yield considerable survival benefits. Given the on-going debate in the scientific community about the value of costly cancer medications [34], and the need to identify cost-effective means of improving cancer treatment and patient outcomes [1], the value and importance of highly cost-effective interventions such as smoking cessation therapy, that have the potential to benefit a great number of cancer patients, cannot be understated.

Despite our evidence of lower hazard of mortality in the sub-group of patients who visited the clinic to receive care and were able to abstain, our findings also revealed that a substantial proportion of cancer patients are not seen at the smoking cessation clinic. This is in-line with what has been reported in other cancer centers [21]. Specifically over the study period, only 21% of our patient sample were seen at least once in the smoking cessation clinic. There was a progressive but slow increase in the percentage of patients seen at our clinic over 8 years, growing from 14.8% of patients diagnosed in 2009 to 34.6% of patients diagnosed in 2016. This increase in referrals over time may in part be attributed to a gradual change in the work culture at KHCC as a result of efforts by smoking cessation clinic staff to continually promote smoking cessation services to cancer practitioners, and due to the designation [by the Joint Commission International (JCI)] of smoking cessation counseling receipt as a performance measure which the Center needed to enhance. Nevertheless, the overall proportions of patients seen at the clinic still remained low even in the more recent years. Certain clinical and demographic factors appeared to be associated with not receiving care at the cessation clinic. These included female sex, having an advanced stage of cancer, and having a non-tobacco related cancer. The significance of these factors confirms the need to promote smoking cessation care as a clinical service with potential value for *all* cancer sites being treated, regardless of clinical as well as demographic characteristics. We have previously reported that a substantial proportion of cancer care providers do not refer cancer patients to the smoking cessation clinic, and that knowledge gaps among these providers exist with regard to the detrimental outcomes associated with continued tobacco use after a cancer diagnosis [35]. Our data thus validate our previous findings and underscore the need for oncology-specific smoking cessation training sessions capable of bridging such knowledge gaps. Availing such a service becomes more urgent when considering that even in subgroups where tobacco cessation may be of higher importance (for example among breast cancer or Hodgkin’s disease patients receiving radiation), the majority of patients were not seen at the smoking cessation clinic (results not presented).

Our analysis was an observational retrospective one using cancer registry and clinic records, and data limitations existed. Despite controlling for several clinical and demographic characteristics that may impact survival, unobservable or residual bias cannot be completely ruled out. For example, socioeconomic status indicators were not available in the Center’s registry data and thus could not be accounted for in our survival analysis. However, in a sub-group of patients with available income and educational status information (namely those visiting the smoking cessation clinic), neither income nor educational status significantly impacted mortality in the short-run (results not presented). We also could not include more detailed information that could have enriched our analysis, such as information on specific chemotherapy regimens being used; and clinical outcomes other than survival (such as hospitalizations, treatment complications). Furthermore, while cigarette smoking status is documented in patient records, we did not have information on use of other forms of tobacco such as the waterpipe, nor did we have information about the smoking status over time of patients who did not visit the smoking cessation clinic, or their receipt of other smoking cessation interventions that were not based at the Center. However, KHCC operates the only fully equipped, evidence-based smoking cessation clinic in the country, offering both pharmacotherapy and intensive counseling. Although the Jordanian Ministry of Health runs three clinics in the country, these are under-equipped in both staff and medications, and comprehensive treatment is not availed through them. [36] Receipt of other smoking cessation interventions beyond the Center was therefore unlikely, particularly because cancer patients at KHCC are covered to receive all cancer-related services (including smoking cessation services) at the Center. Thus, the relapse rates for non-clinic patients (if quit attempts were even made), were likely to have been unassisted and therefore considerably higher than when receiving specialized smoking cessation care: quit rates in cancer patients in Jordan – if they are left to self-manage – are likely lower than the rates reported in published studies [37–39]. This is because smoking is a socially accepted and normalized behavior in the country and Jordanians are high [per capita] consumers of cigarettes; compliance with smoke-free policies is poor and secondhand smoke exposure is high; and few resources are available for smokers who want to quit [40–43]. It is therefore highly plausible that the group of non-clinic patients in our study represented cancer patients who either continued to smoke or attempted to quit alone using minimal evidence-based resources. Finally, we controlled for various confounding factors when analyzing our data to ensure that patients receiving smoking cessation care and those who did not receive any clinic care, were otherwise comparable. Thus, it is reasonable to suggest that the receipt of specialized smoking cessation care was influencing abstinence, which in turn led to lower risk of death.

In summary, our results demonstrate the short-term survival value of clinic-assisted abstinence in cancer patients who were smokers on diagnosis, thus underscoring the importance of availing smoking cessation services to assist smokers in achieving abstinence. Our findings also support the need to heavily promote and increase the receipt of smoking cessation care in cancer patients. Because we address a diverse ‘real-world’ cancer patient population, our results have the potential to resonate with oncology practitioners.

## Conclusions

Our study on Jordanian cancer patients at KHCC who smoked demonstrates that smoking cessation achieved through specialized smoking cessation care (pharmacotherapy and counseling) can yield survival benefits for patients within the short-run (during cancer treatment), and emphasizes the need for availing and promoting evidence-based smoking cessation interventions to cancer patients as a means of integrating in oncology practice a cost-effective tool for improving patient outcomes.

## Data Availability

The datasets used and/or analyzed during the current study are available from the corresponding author on reasonable request.

## Abbreviations

KHCC: King Hussein Cancer Center.

## References

[1] Prager GW, Braga S, Bystricky B, Qvortrup C, Criscitiello C, Esin E, Sonke GS, Martinez GA, Frenel JS, Karamouzis M (2018). Global cancer control: responding to the growing burden, rising costs and inequalities in access. ESMO open.

[2] Karam-Hage M, Cinciripini PM, Gritz ER (2014). Tobacco use and cessation for cancer survivors: an overview for clinicians. CA Cancer J Clin.

[3] Gritz ER, Toll BA, Warren GW (2014). Tobacco use in the oncology setting: advancing clinical practice and research. Cancer Epidemiol Biomarkers Prev.

[4] Land SR, Toll BA, Moinpour CM, Mitchell SA, Ostroff JS, Hatsukami DK, Duffy SA, Gritz ER, Rigotti NA, Brandon TH (2016). Research priorities, measures, and recommendations for assessment of tobacco use in clinical Cancer research. Clin Cancer Res.

[5] Burcu M, Steinberger EK, Sorkin JD (2016). Health care access and smoking cessation among cancer survivors: implications for the affordable care act and survivorship care. J Cancer Surviv.

[6] Ostroff JS, Goffin JR, Khuri FR, Warren GW (2016). Perspective on the National Comprehensive Cancer Network's clinical practice guidelines for smoking cessation. J Oncol Pract.

[7] Fiore MC, Adsit R (2016). Will hospitals finally "do the right thing"? Providing evidence-based tobacco dependence treatments to hospitalized patients who smoke. Jt Comm J Qual Patient Saf.

[8] Lucchiari C, Masiero M, Botturi A, Pravettoni G (2016). Helping patients to reduce tobacco consumption in oncology: a narrative review. SpringerPlus.

[9] Chang EHE, Braith A, Hitsman B, Schnoll RA (2017). Treating nicotine dependence and preventing smoking relapse in Cancer patients. Expert Rev Qual Life Cancer Care.

[10] Kaiser Emily G., Prochaska Judith J., Kendra Matthew S. (2018). Tobacco Cessation in Oncology Care. Oncology.

[11] Crivelli JJ, Xylinas E, Kluth LA, Rieken M, Rink M, Shariat SF (2014). Effect of smoking on outcomes of urothelial carcinoma: a systematic review of the literature. Eur Urol.

[12] Florou AN, Gkiozos IC, Tsagouli SK, Souliotis KN, Syrigos KN (2014). Clinical significance of smoking cessation in subjects with cancer: a 30-year review. Respir Care.

[13] McCarter K, Martinez U, Britton B, Baker A, Bonevski B, Carter G, Beck A, Wratten C, Guillaumier A, Halpin SA (2016). Smoking cessation care among patients with head and neck cancer: a systematic review. BMJ Open.

[14] Hawari FI, Nazer LH, Addassi A, Rimawi D, Jamal K (2016). Predictors of ICU admission in patients with Cancer and the related characteristics and outcomes: a 5-year registry-based study. Crit Care Med.

[15] Curtis A, Ondracek RP, Murekeyisoni C, Kauffman E, Mohler J, Marshall J (2017). Tobacco use and outcome in radical prostatectomy patients. Cancer Med.

[16] Sharp L, McDevitt J, Brown C, Carsin AE, Comber H (2017). Association between smoking at diagnosis and cause-specific survival in patients with rectal cancer: results from a population-based analysis of 10,794 cases. Cancer.

[17] Tabuchi T, Goto A, Ito Y, Fukui K, Miyashiro I, Shinozaki T (2017). Smoking at the time of diagnosis and mortality in cancer patients: what benefit does the quitter gain?. Int J Cancer.

[18] Tamakoshi A, Nakamura K, Ukawa S, Okada E, Hirata M, Nagai A, Matsuda K, Kamatani Y, Muto K, Kiyohara Y (2017). Characteristics and prognosis of Japanese colorectal cancer patients: the BioBank Japan project. J Epidemiol.

[19] Japuntich Sandra J, Kumar Pallavi, Pendergast Jane F, Juarez Caballero Grelda Yazmin, Malin Jennifer L, Wallace Robert B, Chrischilles Elizabeth A, Keating Nancy L, Park Elyse R (2018). Smoking Status and Survival Among a National Cohort of Lung and Colorectal Cancer Patients. Nicotine & Tobacco Research.

[20] Ordonez-Mena JM, Walter V, Schottker B, Jenab M, O'Doherty MG, Kee F, Bueno-de-Mesquita B, Peeters PHM, Stricker BH, Ruiter R (2018). Impact of prediagnostic smoking and smoking cessation on colorectal cancer prognosis: a meta-analysis of individual patient data from cohorts within the CHANCES consortium. Ann Oncol.

[21] Goldstein AO, Ripley-Moffitt CE, Pathman DE, Patsakham KM (2013). Tobacco use treatment at the U.S. National Cancer Institute's designated Cancer centers. Nicotine Tob Research.

[22] Warren GW, Marshall JR, Cummings KM, Toll BA, Gritz ER, Hutson A, Dibaj S, Herbst R, Mulshine JL, Hanna N (2013). Addressing tobacco use in patients with cancer: a survey of American Society of Clinical Oncology members. J Oncol Pract.

[23] Obeidat NA, Ayub HS, Amarin R, Aburajab Altamimi B, Ghonimat I, Abughosh S, Hawari FI (2016). Smoking cessation support among oncology practitioners in a regional Cancer Center in the Middle East-Improving a critical Service for Cancer Care. Oncologist.

[24] Peters EN, Warren GW, Sloan JA, Marshall JR (2016). Tobacco assessment in completed lung cancer treatment trials. Cancer.

[25] King Hussein Cancer Foundation. King Hussein Cancer Center http://www.khcc.jo/. Accessed 4 June 2019.

[26] Cheung KL, de Ruijter D, Hiligsmann M, Elfeddali I, Hoving C, Evers S, de Vries H (2017). Exploring consensus on how to measure smoking cessation. A Delphi study. BMC Public Health.

[27] Velicer WF, Prochaska JO (2004). A comparison of four self-report smoking cessation outcome measures. Addict Behav.

[28] Tran BT, Halperin A, Chien JW (2011). Cigarette smoking and outcomes after allogeneic hematopoietic stem cell transplantation. Biol Blood Marrow Transplant.

[29] Ehlers SL, Gastineau DA, Patten CA, Decker PA, Rausch SM, Cerhan JR, Hogan WJ, Ebbert JO, Porrata LF (2011). The impact of smoking on outcomes among patients undergoing hematopoietic SCT for the treatment of acute leukemia. Bone Marrow Transplant.

[30] Gajdos C, Hawn MT, Campagna EJ, Henderson WG, Singh JA, Houston T (2012). Adverse effects of smoking on postoperative outcomes in cancer patients. Ann Surg Oncol.

[31] Steinberger E, Kollmeier M, McBride S, Novak C, Pei X, Zelefsky MJ (2015). Cigarette smoking during external beam radiation therapy for prostate cancer is associated with an increased risk of prostate cancer-specific mortality and treatment-related toxicity. BJU Int.

[32] Lugg ST, Tikka T, Agostini PJ, Kerr A, Adams K, Kalkat MS, Steyn RS, Rajesh PB, Bishay E, Thickett DR (2017). Smoking and timing of cessation on postoperative pulmonary complications after curative-intent lung cancer surgery. J Cardiothorac Surg.

[33] Zhang P, Nie X, Bie Z, Li L (2018). Impact of heavy smoking on the benefits from first-line EGFR-TKI therapy in patients with advanced lung adenocarcinoma. Medicine.

[34] Cohen D (2017). Cancer drugs: high price, uncertain value. BMJ.

[35] Obeidat NA, Hawari FI, Amarin R, Altamimi BA, Ghonimat IM (2017). Educational needs of oncology practitioners in a regional Cancer Center in the Middle East-Improving the content of smoking cessation training programs. J Cancer Educ.

[36] Ayub H, Obeidat N, Leischow S, Glynn T, Hawari F (2016). Jordan tobacco dependence treatment guidelines: rationale and development. East Mediterr Health J.

[37] Simmons VN, Litvin EB, Jacobsen PB, Patel RD, McCaffrey JC, Oliver JA, Sutton SK, Brandon TH (2013). Predictors of smoking relapse in patients with thoracic cancer or head and neck cancer. Cancer.

[38] Hopenhayn Claudia, Christian W. Jay, Christian Amy, Studts Jamie, Mullet Timothy (2013). Factors associated with smoking abstinence after diagnosis of early stage lung cancer. Lung Cancer.

[39] Alton D, Eng L, Lu L, Song Y, Su J, Farzanfar D, Mohan R, Krys O, Mattina K, Harper C (2018). Perceptions of continued smoking and smoking cessation among patients with Cancer. J Oncol Pract.

[40] Jaghbir M, Shareif S, Ahram M (2014). Quitting smoking and utilization of smoking cessation services in Jordan: a population-based survey. East Mediterr Health J.

[41] WHO report on the global tobacco epidemic, 2017. Country Profile: Jordan Retrieved from http://www.who.int/tobacco/surveillance/policy/country_profile/jor.pdf?ua=1. Accessed 4 June 2019.

[42] The tobacco atlas. Consumption. Retrieved from https://tobaccoatlas.org/topic/consumption/. Accessed 4 June 2019.

[43] Jordanian Department of Statistics (2010). Smoking in Jordan.

